# Most degenerative cervical myelopathy remains undiagnosed, particularly amongst the elderly: modelling the prevalence of degenerative cervical myelopathy in the United Kingdom

**DOI:** 10.1007/s00415-022-11349-8

**Published:** 2022-09-02

**Authors:** Ben Grodzinski, Daniel J. Stubbs, Benjamin M. Davies

**Affiliations:** 1 Department of Intensive Care, Royal Sussex County Hospital, University Hospitals Sussex NHS Foundation Trust, Eastern Road, Brighton, UK; 2 University Division of Anaesthesia, Department of Medicine, Addenbrooke’s Hospital, Hills Road, Cambridge, UK; 3 Division of Neurosurgery, Department of Clinical Neurosciences, University of Cambridge, Cambridge, UK

**Keywords:** Degenerative, Cervical, Myelopathy, Spondylotic, Epidemiology, Prevalence, Incidence, Data science

## Abstract

**Background:**

Degenerative cervical myelopathy (DCM) is a poorly recognised form of spinal cord injury which arises when degenerative changes in the cervical spine injure the spinal cord. Timely surgical intervention is critical to preventing disability. Despite this, DCM is frequently undiagnosed, and may be misconstrued as normal ageing. For a disease associated with age, we hypothesised that the elderly may represent an underdiagnosed population. This study aimed to evaluate this hypothesis by comparing age-stratified estimates of DCM prevalence based on spinal cord compression (SCC) data with hospital-diagnosed prevalence in the UK.

**Methods:**

We queried the UK Hospital Episode Statistics database for admissions with a primary diagnosis of DCM. Age-stratified incidence rates were calculated and extrapolated to prevalence by adjusting population-level life expectancy to the standardised mortality ratio of DCM. We compared these figures to estimates of DCM prevalence based on the published conversion rate of asymptomatic SCC to DCM.

**Results:**

The mean prevalence of DCM across all age groups was 0.19% (0.17, 0.21), with a peak prevalence of 0.42% at age 50–54 years. This contrasts with estimates from SCC data which suggest a mean prevalence of 2.22% (0.436, 2.68) and a peak prevalence of 4.16% at age > 79 years.

**Conclusions:**

To our knowledge, this is the first study to estimate the age-stratified prevalence of DCM and estimate underdiagnosis. There is a substantial difference between estimates of DCM prevalence derived from SCC data and UK hospital activity data. This is greatest amongst elderly populations, indicating a potential health inequality.

**Supplementary Information:**

The online version contains supplementary material available at 10.1007/s00415-022-11349-8.

## Introduction

Degenerative cervical myelopathy (DCM) is a progressive neurological condition which occurs when degenerative changes of the cervical spine, such as disc prolapse, osteophyte formation or ligament thickening, stress and injure the spinal cord [1]. This can lead to a variety of symptoms throughout the body [2, 3]. Initially, these are often mild or subclinical, but with time progress in severity and number. Loss of dexterity and numbness, imbalance, falls, and pain are the focus of current clinical measurement tools [4].

Diagnosing DCM has proven difficult, with most patients facing years of worsening symptoms before the condition is recognised [5]. This has consequences for treatment and outcomes [6]. Surgery to remove the mechanical stress on the spinal cord is currently the only disease modifying therapy. Although not required in all circumstances, with a period of watchful waiting an option in mild and stable forms of the disease, for those requiring surgery, its effectiveness is dependent on length of prior symptoms [7]. Today, therefore, disability from DCM is high, with ~ 40% left unable to return to work and ~ 50% dependent on others [6]. In a study comparing quality of life amongst chronic disease, people with DCM [8] had amongst the worst quality-of-life scores [9]. Consequently, facilitating early diagnosis and timely treatment are critical priorities.

Whilst delays exist, extrapolations from natural history studies have suggested that most patients will never get a diagnosis. These studies have conducted longitudinal follow-up on individuals with asymptomatic spinal cord compression (SCC), a common finding on magnetic resonance imaging (MRI) which leads to the development of DCM in a subset of people. A meta-analysis of these studies has estimated the population prevalence of DCM at 2.3% [10], which starkly contrasts known epidemiology [11]. Identifying groups with prominent underdiagnosis would facilitate aims to offer timely surgery.

Radiologically, the occurrence of degenerative spinal changes, and so the prevalence of radiologically-defined SCC, increases with age [12]. Logic would therefore suggest so too would the occurrence of DCM. However, this has not so far not been demonstrated in the literature. In a recent report for Myelopathy.org, a DCM charity, hospital admission in England and Wales peaked at age 70 before falling in older age groups [13].

Given our ageing population, and the recognition that DCM severity is associated with age [14], the possibility of underdiagnosis represents an important uncertainty [15]. The age-stratified prevalence of DCM in the UK has not been previously characterised. This study aimed to evaluate this by comparing estimated population prevalence from SCC data with hospital-diagnosed prevalence in the UK, stratifying prevalence by age. Underdiagnosis would be one explanation for any discrepancy between these figures. We hypothesised that discrepancies between these two measures would be more common in older age groups.

## Methods

### Estimated prevalence: UK hospital data

To estimate the prevalence of DCM in the United Kingdom (UK), we analysed the Hospital Episode Statistics (HES) database. This is made up of several datasets, detailing all admissions, accident and emergency attendances, and outpatient appointments at National Health Service (NHS) hospitals in England [16]. However, only the Hospital Admitted Patient Care Activity dataset, detailing admissions, provides data stratified by both diagnostic code and age [17].

The dataset was searched for all years for which age-stratified data were available, from 2012–2013 to 2018–2019. The ICD-10 codes most compatible with the definition of DCM were M47.1, M50.0, M99.3, M99.4, and M99.5. The ‘primary diagnosis’ values were used, aiming to select for patients in hospital specifically for DCM, rather than with DCM.

Within the dataset, age-stratified values are provided for ‘Finished Consultant Episodes’ (FCEs), which reflect a patient’s hospital admission under a single lead consultant. Thus, the number of FCEs (and subsequent calculations) may differ from the number of admissions, since a single admission may change lead consultant. For the years and ICD-10 codes queried, there was a total of 35,078 FCEs and 28,517 admissions for DCM.

Cases of DCM were totalled by age group and year. Age-stratified incidence rates (per 100,000 person years) were calculated using the published population data for England available from the Office for National Statistics (ONS) [18].

We modelled prevalence as *P* = *ID* where *I* = the estimated incidence rate and *D* is the mean duration of disease. This was performed by 5-year age strata. Since the underlying disease process of DCM is incurable, the duration of disease was taken to be equal to age-specific life expectancy. This was calculated by adjusting age-specific population-level life expectancy from the Office for National Statistics (ONS) [19] to the Standardised Mortality Ratio (SMR) of 1.18 in DCM [20], using established life-table techniques [21, 22]. Full details of these calculations are available in the supplementary material.

### Estimated prevalence: extrapolation from spinal cord compression data

We next estimated prevalence of DCM based on published data on the age-stratified prevalence of SCC and rates of conversion from SCC to DCM.

In the absence of a UK-based study, we used the age-stratified prevalence of SCC from a high-quality 2012 cross-sectional study of a healthy population in Japan [12] using MRI-defined markers.

A recent meta-analysis which included the above study estimated the pooled prevalence of both SCC and DCM [10]. Since SCC is necessary but not sufficient for DCM, this allows calculation of a ‘conversion rate’, the proportion of people with SCC who also have DCM. The meta-analysis estimated the pooled conversion rate from all studies identified, and also provided the possible upper and lower bounds of the conversion rate from individual studies. We used this information to estimate upper and lower bounds of DCM prevalence as well as an estimate based on the meta-analysed rate of conversion.

### Comparing prevalence estimates

Estimates were reformatted into standardised age groups, to allow graphical, numerical, and statistical comparison. 95% confidence intervals for the observed prevalence were calculated according to standard methodology [23].

### Investigating mismatch between prevalence estimates

To investigate the possibility of a cohort effect accounting for the difference between our two prevalence estimates, cohort graphs were produced [24]. These investigate whether the ‘birth cohort’ (i.e., year of birth) affects prevalence independently of age. To produce these, the mid-point of age for each age group in the data was subtracted from the year of survey to calculate the year of birth. Year of birth was then plotted against age and prevalence.

To investigate the possibility of case ascertainment bias due to the use of inpatient-only hospital data, a preliminary analysis of outpatient attendances was also conducted. Using the HES outpatients dataset [25], we calculated the total number of attendances for DCM, using the same years and ICD-10 codes as for the inpatient analysis. Age stratification of outpatient attendances was not possible as this information is not publicly available. Mean incidence of outpatient attendances was then calculated using the same ONS population data as for the inpatient analysis. Since data on the age of attendees were not available, life expectancy and outpatient DCM prevalence could not be calculated.

### Data analysis and presentation

All data analysis was performed using R version 3.6.1 [26]. All data and R scripts are available as supplementary materials. Data are presented as mean (± Standard Deviation) or as calculated prevalence or incidence with [95% Confidence Interval].

## Results

### Estimated DCM prevalence: UK hospital data

**Fig. 1:**
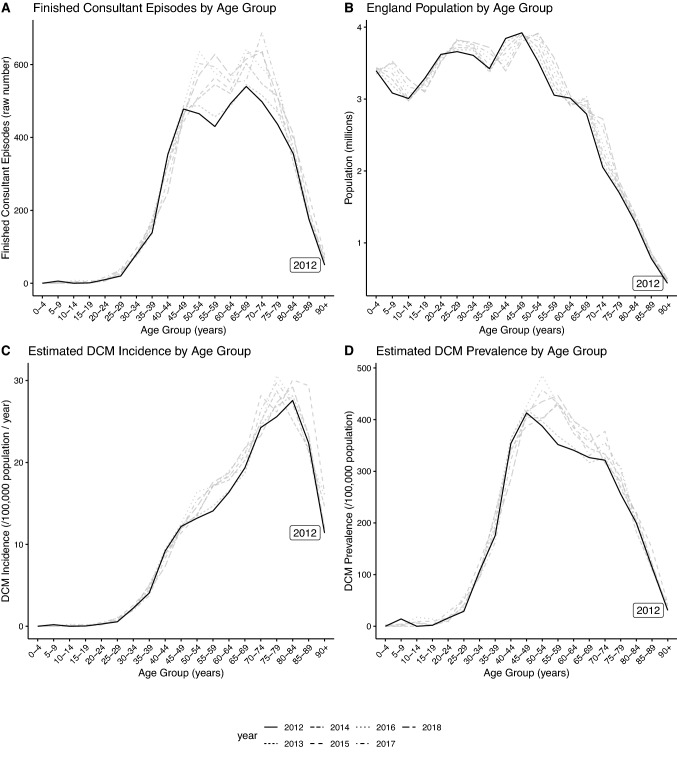
Estimated DCM prevalence using Hospital Episode Statistics (HES) data. In each panel, the data from 2012 are highlighted as black lines, with other years shown as grey lines for comparison. Epidemiological parameters are shown on the vertical axis, with age group on the horizontal axis

The number of FCEs, and the calculated incidence and prevalence of DCM are shown in Fig. 1. Values from 2012 are highlighted to facilitate comparison, since this is the closest year to the SCC data from which estimated DCM prevalence is derived.

The age group with the greatest number of FCEs was 65–69 years (mean 590 ± 42.4 FCEs/year). However, peak incidence occurred in the 75–79 year age group (mean 27.9 ± 1.89 FCEs/100,000 population/year). Using SMR-adjusted life expectancy as the estimate of disease duration in each age group, peak prevalence occurred in those aged 50–54 years with a prevalence of 0.42% [0.41, 0.44]. The mean prevalence across all age groups was 0.19% (± 0.016). Age-stratified prevalence values are shown in Table 1.

**Table 1:** Estimated age-stratified prevalence of degenerative cervical myelopathy (DCM) using hospital episode statistics (HES) data for 2012–2019

Age (years)	Estimated DCM prevalence using HES data (% population)	
	Lower bound of 95% CI	Upper bound of 95% CI
0–4	0.00016	0.0026
5–9	0.0031	0.0081
10–14	0.0038	0.0094
15–19	0.003	0.0077
20–24	0.012	0.019
25–29	0.038	0.049
30–34	0.096	0.11
35–39	0.18	0.21
40–44	0.33	0.35
45–49	0.4	0.43
50–54	0.41	0.44
55–59	0.4	0.43
60–64	0.36	0.39
65–69	0.33	0.36
70–74	0.33	0.35
75–79	0.27	0.29
80–84	0.2	0.21
85–89	0.11	0.13
Above 90	0.033	0.04

### Estimated DCM prevalence: SCC prevalence data

**Fig. 2:**
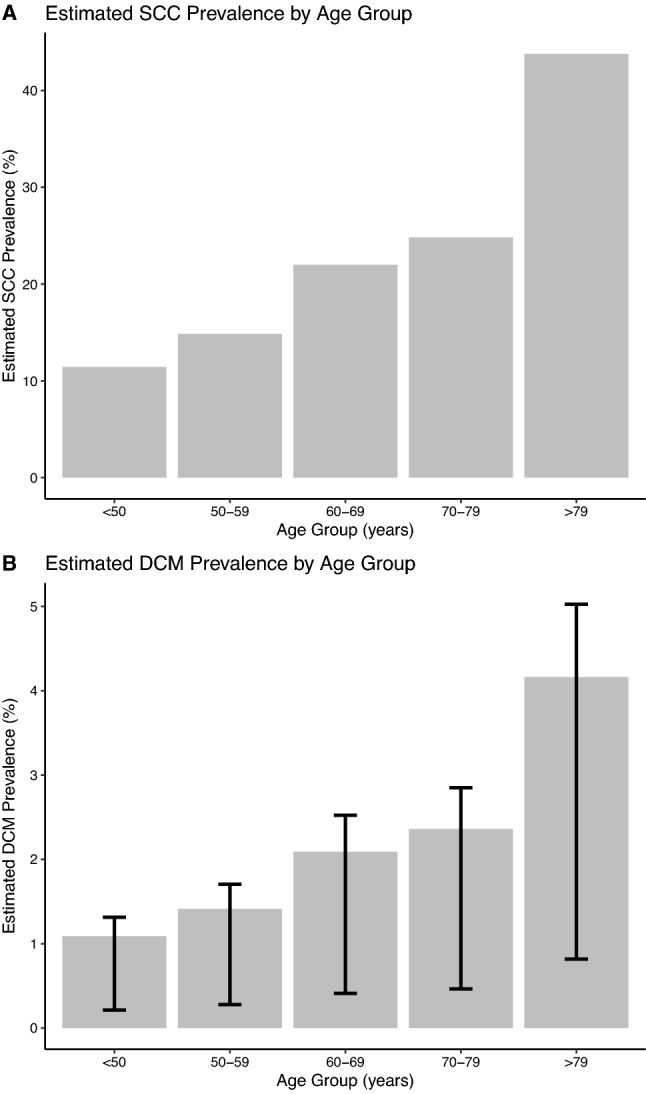
Estimated DCM prevalence using SCC data. Panel **A** Previously published prevalence estimates of SCC reproduced using data from [12]. Panel **B** Estimated DCM prevalence, as a product of SCC prevalence and the published rate of co-occurrence of SCC with DCM from [10]. Column height indicates the meta-analysed conversion rate, and error bars indicate the upper and lower bounds of this conversion rate

The previously reported age-stratified prevalence of SCC [12] is shown in Fig. 2A, and the predicted prevalence of DCM in Fig. 2B. In panel B, column height corresponds to estimates generated from the pooled conversion rate of SCC to DCM, whilst error bars correspond to the upper and lower bounds of the conversion rate.

The age group with the highest estimated prevalence of DCM using this method was those aged over > 79 years (4.16% using meta-analysed estimate, 5.03% upper, 0.82% lower). Prevalence rose with age group (Fig. 2). The mean prevalence across all age groups was 2.22% (2.68% upper, 0.436% lower).

### Comparing prevalence estimates

**Fig. 3:**
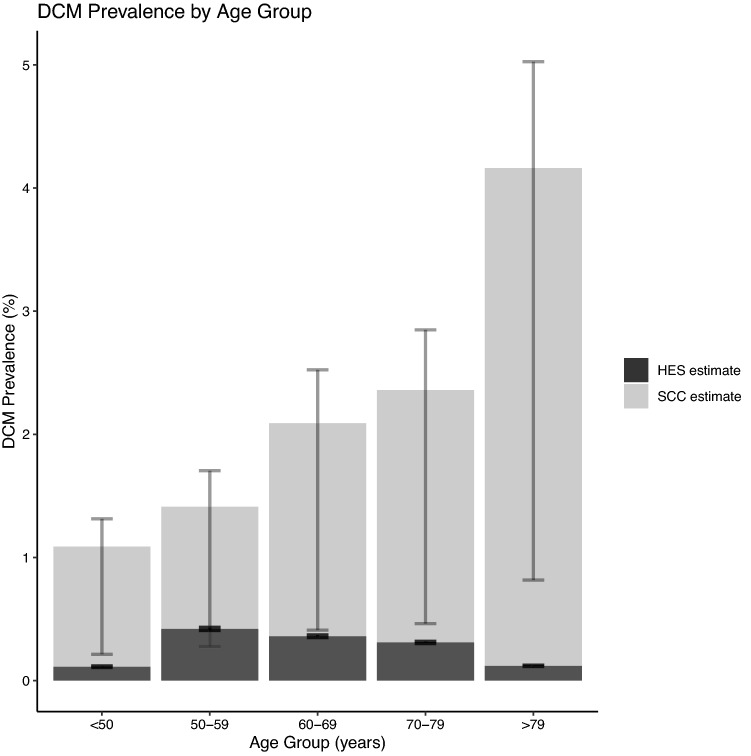
Comparing DCM prevalence estimates. Estimates derived from the conversion rate of SCC to DCM are shown in light grey, with error bars indicating the upper and lower bounds of this conversion rate. Estimates derived from HES data are shown in dark grey, with error bars indicating 95% confidence intervals

Estimates of DCM prevalence using both methods are shown in Table 2 and plotted in Fig. 3. The upper and lower bounds of these estimates overlap only in the 50–59 age group.

**Table 2:** Comparison of DCM prevalence estimates using hospital-derived and SCC-derived data

Age	Hospital-derived prevalence (± 95% CI)	SCC-derived prevalence (upper and lower bounds)
< 50	0.112 (0.120, 0.106)	1.088 (1.314, 0.213)
50–59	0.419 (0.432, 0.406)	1.412 (1.705, 0.277)
60–69	0.360 (0.372, 0.349)	2.090 (2.524, 0.410)
70–79	0.310 (0.320, 0.300)	2.359 (2.848, 0.463)
> 79	0.120 (0.126, 0.114)	4.162 (5.025, 0.816)

### Cohort analysis

**Fig. 4:**
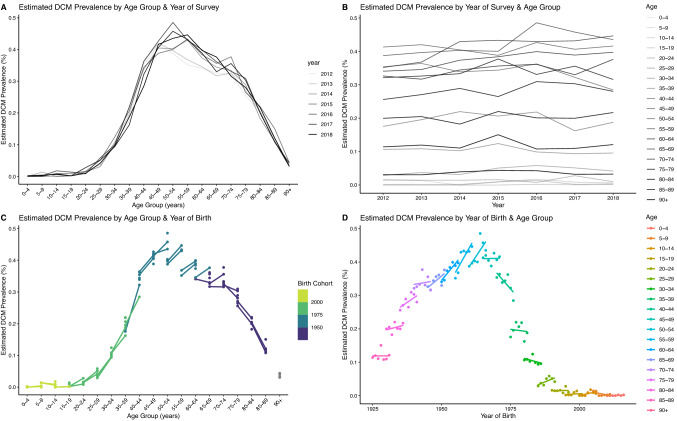
Estimated DCM prevalence by age, year, age group, and of birth. Estimates use HES data from 2012–2019

One potential explanation for the age-dependent difference in estimated prevalence of DCM using these two methods is a cohort effect. We explored this hypothesis by generating plots of prevalence against age, year of survey, and cohort/year of birth, as shown in Fig. 4.

Prevalence is strongly affected by age (panel A), and there is relatively little variation by year of survey (panel B). The relationship between age and prevalence is affected by birth cohort: in panel C, lines representing different birth cohorts spanning the same age groups are not parallel, whilst in panel D, gradients of the lines representing prevalence vs year of birth within a single age group are non-zero. Because the years of survey (2012–2019) are narrow compared to the years of birth (1925–2019), many age groups do not have overlapping cohorts, making assessment of a cohort effect specifically in the > 75 age group difficult.

### Outpatient analysis

Another potential explanation for the mismatch between prevalence estimates is case ascertainment bias due to the inpatient-only nature of the HES database. To explore this, we conducted a preliminary analysis of outpatient data [25], for which age stratification is not publicly available.

The mean incidence of outpatient attendances with an ICD-10 code corresponding to DCM was 1.19 attendances/100,000 population/year. This compares to a mean inpatient incidence of 11.3 FCEs/100,000 population/year.

## Discussion

In this study, we provided the first estimates of the age-stratified prevalence of DCM using two data sources—UK hospital admissions (HES) data, and published spinal cord compression (SCC) prevalence data. There was a mismatch in estimates using these two measures, with estimates from HES data lower than those from SCC data in all age groups other than 50–59 years (Table 2, Fig. 3). This mismatch is particularly pronounced in the oldest age group, > 79 years.

### A mismatch exists between prevalence estimates

A number of explanations could account for the generalised mismatch between prevalence estimates.

First, SCC-derived DCM prevalence may represent an overestimation of true prevalence. This could occur if the conversion rate from SCC to DCM used in this study is greater than the rate in the UK population. However, this conversion rate is derived from a high-quality meta-analysis [10], which includes data from ten different countries across three continents, making the conversion rate an unlikely source of inaccuracy. Overestimation could also occur if the SCC prevalence used in this study, measured in a Japanese population, is higher than the SCC prevalence in the UK population on which the hospital data are based. However, the same meta-analysis found that the prevalence of SCC was in fact higher in American/European populations than Asian populations [10], suggesting that the SCC-derived prevalence may in fact be an underestimate. Hence, overestimation of the SCC-derived prevalence is unlikely to explain the observed mismatch.

Second, hospital-derived prevalence could represent an underestimation of true prevalence. This might have several causes. For instance, ICD-10 coding has a poor sensitivity for DCM [27]—hence, a number of DCM cases are likely to have been missed. However, the ICD-10 codes used in this study are considered to provide the best possible balance of sensitivity against specificity [15]. Inaccuracy in the hospital-derived prevalence estimate could also arise from the inherent assumptions of steady-state populations and average duration of disease when extrapolating from incidence to prevalence, although this is standard methodology for calculating prevalence [22, 28]. Hospital-derived prevalence may actually be an overestimate, since the calculations are based on FCEs rather than admissions, and a single admission may have more than one FCE.

Another potential cause for underestimation in the hospital-derived prevalence is case ascertainment bias. These prevalence estimates are based solely on hospital admissions primarily due to DCM, for instance, for surgical management or in those with severe and emergent symptoms. This is likely a specific but poorly sensitive metric to use in estimating population prevalence, although it may be of benefit to those involved in the planning and delivery of surgical services. By contrast, prevalence derived from studies which recruit healthy volunteers for an MRI scan to detect SCC and neurological exam to detect DCM [12] yielded, as expected, higher estimates of DCM prevalence.

One interpretation of this mismatch is therefore that this latter metric is capturing DCM patients with a milder phenotype, or who are perhaps not undergoing or being offered surgical treatment of their DCM and hence would only be captured using community-based metrics [29]. If our estimate of prevalence using HES data is primarily reflecting surgical management of DCM, then this may also explain the differing trends of prevalence by age (Fig. 3) as rates of surgery are higher in those who are still of working age [30], despite older DCM patients having more severe disease [14].

However, case ascertainment bias due to our inpatient-only analysis is unlikely to fully explain the mismatch—preliminary analysis of outpatient data showed that the mean incidence of outpatient attendances for DCM was just over a tenth the incidence of inpatient FCEs. Hence, even adding outpatient attendances, the mismatch remains. Previous estimation of DCM prevalence using primary care data has also yielded an overall prevalence of 0.04% [29], a fifth of our estimate. Although the HES database does not include primary care data, the diagnosis of DCM requires secondary care assessment [5], so this is unlikely to be a substantial source of case ascertainment bias.

Having explored the above explanations, we are left with the possibility that the prevalence mismatch represents underdiagnosis across the population. Awareness of DCM amongst both patients and healthcare professionals is also poor [31], which may lead to delayed diagnosis [6]. This interpretation is also supported by our finding of a widening diagnostic gap with age, discussed below.

### Hospitalised DCM prevalence declines in older age groups

In addition to the generalised mismatch between prevalence estimates, we found differing trends in prevalence in older age groups. SCC-derived estimates showed increasing prevalence in the oldest age groups, whilst HES-derived estimates showed decreasing prevalence (Table 2, Fig. 3). This potentially separate phenomenon also has several possible explanations.

First, older patients may be less likely to be hospitalised for DCM, and instead may be living with DCM and thus absent from our estimates using these metrics. Hospital admissions for a different problem, where the patient also has DCM, would not be included in the observed prevalence values, since these are based on primary hospital diagnosis. Reduced hospital admissions for DCM in older age groups could represent a health inequality—older DCM patients may be considered ‘not suitable for surgery’ due to the presence of comorbidities or frailty. However, such an inequality would be against management guidance for DCM [7], and hence is unlikely to provide a full explanation.

Second, low hospital prevalence in older age groups may reflect a cohort effect, i.e., an effect of year of birth that is independent of age. Hypothetically, those age > 75 at the time of these HES data may have had limited access to MRI investigation as younger adults, since MRI scans were not in routine use in the NHS until the 1980s [32], meaning that potentially fewer cases were picked up. To investigate this hypothesis, we generated cohort graphs using the available data (Fig. 4). Different birth cohorts have different observed prevalence over the same age ranges, but the narrow range of years for which HES data is available and the potential impact of case ascertainment bias already described means that this hypothesis can be neither confirmed nor refuted. Data from a wider time period and more sensitive measures of case ascertainment would be required to fully explore this hypothesis. However, on an empirical level, a cohort effect is unlikely to fully account for the differing trends—as a progressive disease, even cases which were not picked up initially should have been detected later.

Third, the conversion rate from SCC to DCM may be negatively affected by age. SCC-derived prevalence is based on a non-stratified conversion rate, since age-stratified conversion rates from SCC to DCM are not available in the literature. Thus, the discrepancy between our two metrics could reflect a false assumption that this rate of conversion or diagnosis is static across age bands. Whilst possible, this explanation seems unlikely—if anything, the conversion rate is likely to be positively affected by age, given the increased vulnerability of the spinal cord [33] and greater severity of degenerative changes [14] with age.

Finally, underdiagnosis in older age groups may explain the differing trends in prevalence. The signs and symptoms of DCM are non-specific [34] and may be mistaken for ‘getting older’ [1]. For instance, a case–control study of patients presenting with hip fracture found that 18% had previously undiagnosed DCM [35]. Physical findings may also be masked by comorbidities such as diabetic neuropathy [36], which become more common with age. Thus, underdiagnosis may act as a unifying explanation for both the decline in hospitalised DCM prevalence, and the generalised mismatch between estimates.

### Access to timely diagnosis and surgery is critical in DCM

If underdiagnosis is even partially responsible for both the generalised prevalence mismatch and the apparent decline in older age groups, this has potentially serious implications. Delayed diagnosis leads to greater disability [6], so timely access to diagnosis is critically important. Furthermore, our results suggest that underdiagnosis may be more likely in older age groups. Older patients still gain meaningful benefit from surgery [14], which should therefore be performed in cases of moderate and severe DCM [7] regardless of age. Thus, the potential impact of underdiagnosis on both health system utilisation and patient quality of life is highly relevant.

## Conclusion

In this study, we showed a mismatch between the estimated prevalence of DCM on the basis of spinal cord compression rates, and the estimated prevalence in UK hospitals. This mismatch is greater in older age groups. Whilst the cause of the mismatch remains unanswered, underdiagnosis is a highly plausible explanation. Given that surgery remains beneficial in these age groups, this should be urgently addressed.

## Supplementary Information

Below is the link to the electronic supplementary material.

Supplementary file1 (DOCX 32 KB)

Supplementary file2 (XLSX 326 KB)

Supplementary file3 (R 2 KB)

Supplementary file4 (CSV 7 KB)

Supplementary file5 (CSV 1 KB)

Supplementary file6 (R 11 KB)

Supplementary file7 (R 7 KB)

Supplementary file8 (CSV 9 KB)

Supplementary file9 (CSV 9 KB)

Supplementary file10 (CSV 0 KB)

Supplementary file11 (R 1 KB)

Supplementary file12 (R 2 KB)

## References

[1] Davies BM (2018). Degenerative cervical myelopathy. BMJ.

[2] Boerger T (2022). Moving beyond the neck and arm: the pain experience of people with degenerative cervical myelopathy who have pain. Global Spine J.

[3] Davies BM (2022). Outcomes of degenerative cervical myelopathy from the perspective of persons living with the condition: findings of a semistructured interview process with partnered internet survey. Global Spine J.

[4] Yanez Touzet A (2022). Clinical outcome measures and their evidence base in degenerative cervical myelopathy: a systematic review to inform a core measurement set (AO Spine RECODE-DCM). BMJ Open.

[5] Hilton B (2019). Route to diagnosis of degenerative cervical myelopathy in a UK healthcare system: a retrospective cohort study. BMJ Open.

[6] Pope DH (2020). Diagnostic delays lead to greater disability in degenerative cervical myelopathy and represent a health inequality. Spine (Phila Pa 1976).

[7] Fehlings MG (2017). A clinical practice guideline for the management of patients with degenerative cervical myelopathy: recommendations for patients with mild, moderate, and severe disease and nonmyelopathic patients with evidence of cord compression. Global Spine J.

[8] Boerger TF (2020). Patient, sufferer, victim, casualty or person with cervical myelopathy: let us decide our identifier. Integ Healthcare J.

[9] Oh T (2017). Comparing quality of life in cervical spondylotic myelopathy with other chronic debilitating diseases using the short form survey 36-health survey. World Neurosurg.

[10] Smith SS (2021). The prevalence of asymptomatic and symptomatic spinal cord compression on magnetic resonance imaging: a systematic review and meta-analysis. Global Spine J.

[11] Boogaarts HD, Bartels RH (2015). Prevalence of cervical spondylotic myelopathy. Eur Spine J.

[12] Nagata K (2012). Prevalence of cervical cord compression and its association with physical performance in a population-based cohort in Japan: the Wakayama Spine study. Spine.

[13] Phillips R (2020). A review of the data availability for developing of a burden of illness model in DCM.

[14] Grodzinski B (2020). The effect of ageing on presentation, management and outcomes in degenerative cervical myelopathy: a systematic review. Age Ageing.

[15] Goacher E (2022). Hospitalisation for degenerative cervical myelopathy in England: insights from the National Health Service Hospital Episode Statistics 2012 to 2019. Acta Neurochir (Wien).

[16] ; Weblink 1]. Available from: https://digital.nhs.uk/data-and-information/data-tools-and-services/data-services/hospital-episode-statistics

[17] ; Weblink 2]. Available from: https://digital.nhs.uk/data-and-information/publications/statistical/hospital-admitted-patient-care-activity#about-this-publication

[18] ; Weblink 3]. Available from: https://www.ons.gov.uk/peoplepopulationandcommunity/populationandmigration/populationestimates/datasets/populationestimatesforukenglandandwalesscotlandandnorthernireland

[19] ; Weblink 4]. Available from: https://www.ons.gov.uk/peoplepopulationandcommunity/birthsdeathsandmarriages/lifeexpectancies/datasets/nationallifetablesunitedkingdomreferencetables

[20] Davies B Life expectancy reduced in patients with Degenerative Cervical Myelopathy. Global Spine J (**Under Review**)

[21] Cutler SJ, Ederer F (1958). Maximum utilization of the life-table method in analyzing survival. J Chronic Dis.

[22] Yeo JD (1998). Mortality following spinal cord injury. Spinal Cord.

[23] ; Weblink 5]. Available from: https://sphweb.bumc.bu.edu/otlt/MPH-Modules/PH717-QuantCore/PH717_ConfidenceIntervals-OneSample/PH717_ConfidenceIntervals-OneSample5.html

[24] Dobson A (2020). Flexible age-period-cohort modelling illustrated using obesity prevalence data. BMC Med Res Methodol.

[25] ; Weblink 6]. Available from: https://digital.nhs.uk/data-and-information/publications/statistical/hospital-outpatient-activity

[26] Team, R. C. (2019). R: a language and environment for statistical computing.

[27] Khan DZ, Davies BM, Kotter MRN (2020). Spinal research—a field in need of standardization. J Rheumatol.

[28] ; Weblink 10]. Available from: https://sphweb.bumc.bu.edu/otlt/mph-modules/ep/ep713_diseasefrequency/EP713_DiseaseFrequency7.html

[29] MacDonald BK (2000). The incidence and lifetime prevalence of neurological disorders in a prospective community-based study in the UK. Brain.

[30] Badhiwala JH (2019). Efficacy and safety of surgery for mild degenerative cervical myelopathy: results of the AOSpine North America and international prospective multicenter studies. Neurosurgery.

[31] Davies BM (2019). RE-CODE DCM (REsearch Objectives and Common Data Elements for Degenerative Cervical Myelopathy): a consensus process to improve research efficiency in DCM, through establishment of a standardized dataset for clinical research and the definition of the research priorities. Global Spine J.

[32] ; Weblink 11]. Available from: https://www.rbht.nhs.uk/blog/history-magnetic-resonance-imaging-mri

[33] Zdunczyk A (2018). The corticospinal reserve capacity: reorganization of motor area and excitability as a novel pathophysiological concept in cervical myelopathy. Neurosurgery.

[34] Davies BM, Munro CF, Kotter MR (2019). A novel insight into the challenges of diagnosing degenerative cervical myelopathy using web-based symptom checkers. J Med Internet Res.

[35] Radcliff KE (2016). High incidence of undiagnosed cervical myelopathy in patients with hip fracture compared with controls. J Orthop Trauma.

[36] Houten JK, Lenart C (2016). Diabetes and cervical myelopathy. J Clin Neurosci.

